# Case Report: Adult liver transplantation using a long-standing mesocaval shunt as portal inflow in congenital absence of the portal vein with atypical variceal bleeding and refractory shunt encephalopathy

**DOI:** 10.3389/fsurg.2026.1887903

**Published:** 2026-08-14

**Authors:** A. V. Shabunin, M. G. Minina, P. A. Drozdov, O. N. Levina, S. A. Astapovich, D. A. Makeev, D. A. Solomatin, I. I. Kurbanov, A. I. Yurik

**Affiliations:** 1 Botkin Hospital, Moscow, Russia; 2 Department of Surgery, Russian Medical Academy of Continuous Professional Education, Moscow, Russia; 3 I. M. Sechenov First Moscow State Medical University (Sechenov University), Moscow, Russia

**Keywords:** Abernethy malformation, congenital absence of the portal vein, congenital portosystemic shunt, hyperammonemic encephalopathy, intestinal dysmotility, liver transplantation, mesocaval shunt, portal inflow reconstruction

## Abstract

Congenital absence of the portal vein (CAPV) is an exceedingly rare vascular malformation in which mesenteric and splenic venous blood bypasses the liver and drains into the systemic circulation. Because congenital portosystemic drainage usually decompresses the splanchnic venous bed, classical portal hypertension is uncommon, and CAPV typically presents with metabolic and vascular complications rather than variceal bleeding. We report the case of a 36-year-old woman with CAPV that presented atypically in early childhood with esophageal variceal hemorrhage, suggesting functional inadequacy or early loss of the native decompressive pathway. At the age of three years, she underwent H-type mesocaval shunting and remained clinically compensated for more than three decades. She later developed refractory shunt-related hyperammonemic encephalopathy with recurrent functional bowel obstruction, without evidence of mechanical obstruction on repeated imaging and laparotomy. Orthotopic liver transplantation was performed using a graft from a brain-dead donor. Because the native portal vein was absent, portal inflow was reconstructed by an end-to-end shunt-to-portal anastomosis using the long-standing mesocaval shunt, avoiding the need for an interposition venous graft. The postoperative course was complicated by a biliary anastomotic stricture managed with staged endoscopic stenting and by late stenosis of the shunt-to-portal anastomosis on postoperative day 212, successfully treated with balloon angioplasty and portal vein stenting. At the 275-day follow-up, the patient remained clinically stable, with no recurrence of encephalopathy and resolution of intestinal dysmotility. This case highlights a rare adult presentation of CAPV requiring liver transplantation and demonstrates that a long-standing mesocaval shunt may serve as a feasible source of portal inflow when the native portal vein is absent.

## 1 Introduction

Congenital absence of the portal vein (CAPV) is an exceedingly rare developmental anomaly in which the native portal vein fails to form and mesenteric and splenic venous blood drains directly into the systemic circulation, bypassing the liver. In the Morgan–Superina classification, CAPV belongs to the spectrum of congenital portosystemic vascular anomalies. In type I malformations, effective intrahepatic portal perfusion is absent; subtype Ib is characterized by a preserved splenomesenteric confluence draining into the systemic venous system (1–3).

A defining hemodynamic feature of CAPV is diversion of splanchnic venous flow through a congenital portosystemic shunt, resulting in decompression of the splanchnic bed; consequently, classical portal hypertension is not a typical feature of CAPV. The clinical phenotype is therefore dominated by metabolic and vascular complications, including hyperammonemic and shunt-related encephalopathy, focal liver lesions, hepatopulmonary syndrome, and portopulmonary hypertension, whereas variceal bleeding is atypical for the classical disease course (2–4).

Nevertheless, a small number of reports have described CAPV presenting with hematemesis and rupture of esophagogastric varices (5, 6). Such an atypical hemorrhagic onset may suggest functional inadequacy or early loss of the congenital decompressive pathway. If the splanchnic venous bed is not effectively decompressed, a portal hypertensive phenotype may develop and require surgical decompression. In anatomically non-reconstructible portal systems, decompressive portosystemic procedures have historically been used, particularly H-type mesocaval anastomosis with an autologous venous interposition graft. The series reported by Gauthier and Sigalet demonstrated reliable splanchnic decompression, prevention of recurrent variceal bleeding, and satisfactory long-term patency of such shunts (7, 8). However, mesocaval shunting does not restore hepatopetal portal flow and does not correct the underlying vascular anomaly; it should therefore be regarded as a decompressive procedure rather than definitive treatment (3, 8).

CAPV must be distinguished from extrahepatic portal vein obstruction (EHPVO), in which the intrahepatic portal branches and the Rex recessus are preserved, so that physiological revascularization through a meso-Rex bypass is feasible (9, 10). In true CAPV these structures are absent and meso-Rex bypass is anatomically impossible; management therefore depends on the individual anatomy and may require portosystemic shunt closure, decompressive shunting, or liver transplantation (3, 9). In the present patient, imaging confirmation of CAPV excluded EHPVO, and meso-Rex bypass was not a therapeutic consideration.

A long-standing decompressive shunt may provide many years of clinical compensation but can later be complicated by severe shunt-related hyperammonemic encephalopathy. In this setting, liver transplantation may become the only definitive treatment option, particularly in patients with refractory encephalopathy, hepatopulmonary syndrome, portopulmonary hypertension, or focal liver lesions (3, 11, 12). Available experience suggests that systemic manifestations of congenital portosystemic shunts may improve after elimination of the portosystemic shunt (3, 13). In addition to classical metabolic and vascular complications, severe gastrointestinal dysmotility, including functional bowel obstruction, may be biologically plausible in this context. Experimental data indicate that ammonia can modulate enteric neuromuscular transmission (14), and impaired intestinal motility has been described in chronic liver disease (15). However, such manifestations are not currently recognized as typical complications of congenital portosystemic shunts (3, 4).

The main technical challenge of liver transplantation in this setting is not the conventional transplant procedure itself, but reconstruction of adequate portal inflow. Potential sources include the splenomesenteric confluence, a previously created portosystemic shunt, large collateral veins, or interposition venous grafts (11, 16).

We report a rare case of adult liver transplantation for CAPV after childhood H-type mesocaval shunting, in which the long-standing mesocaval shunt was used as the source of portal inflow to the graft. The case is notable for atypical childhood variceal bleeding, late refractory shunt-related hyperammonemic encephalopathy, and severe functional bowel obstruction that regressed after transplantation.

## 2 Case description

A 36-year-old woman was admitted on an emergency basis for progressive shunt-related hyperammonemic encephalopathy.

**Figure 1:**
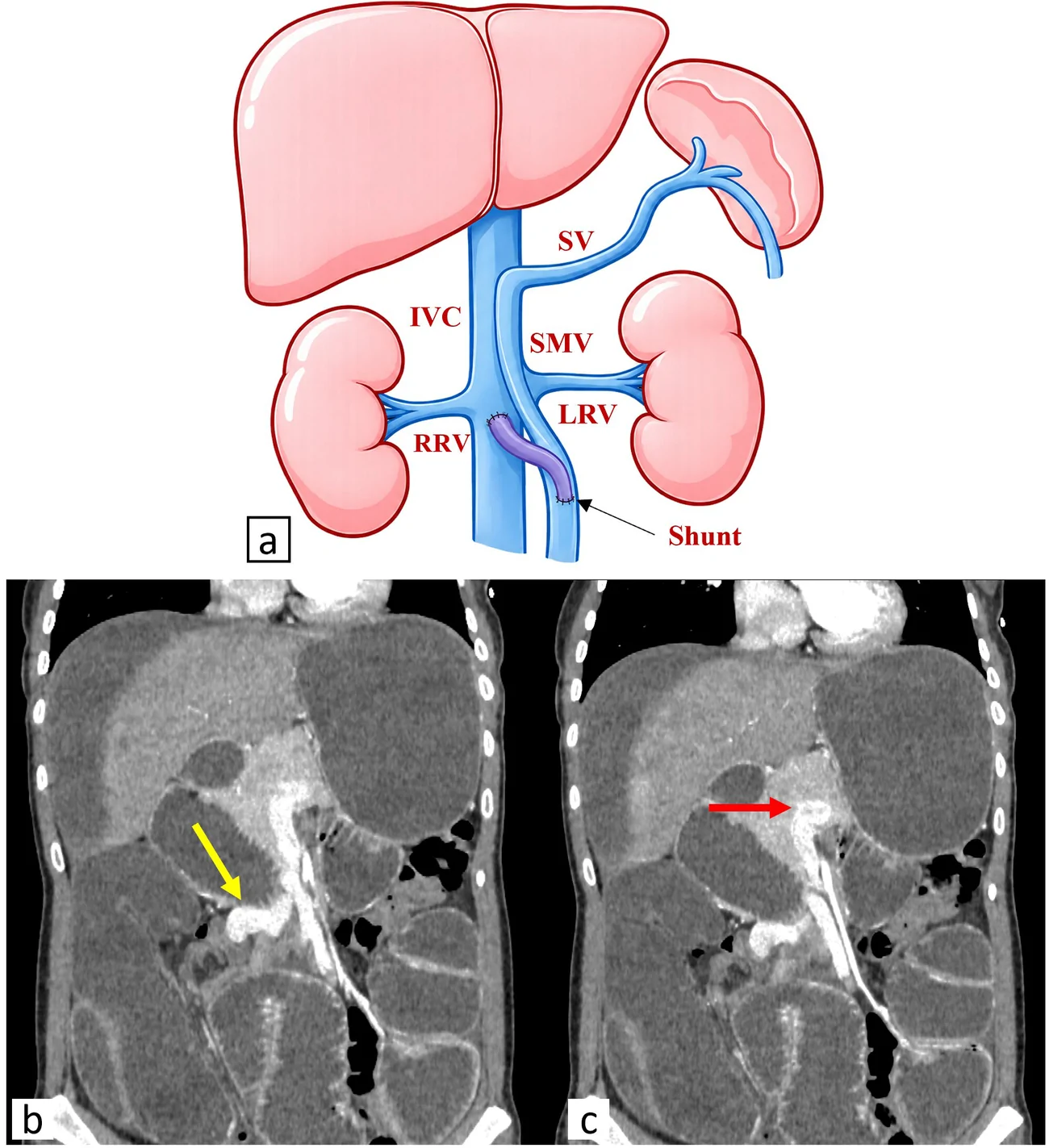
Vascular anatomy of the patient with congenital absence of the portal vein. **(a)** Schematic representation of the H-type mesocaval shunt. **(b)** Multidetector computed tomography (MDCT) visualization of the shunt (yellow arrow). **(c)** MDCT visualization of the spleno-mesenteric confluence in the absence of a portal vein trunk (red arrow). IVC, inferior vena cava; SV, splenic vein; SMV, superior mesenteric vein; RRV, right renal vein; LRV, left renal vein.

At three years of age (1992), she had undergone an H-type mesocaval shunt for CAPV that had presented with bleeding from esophageal varices (Figure 1). Over the following three decades, the patient remained clinically compensated. The first episodes of encephalopathy occurred in 2009, and in 2010 hyperammonemia up to 80 μmol/L (136 μg/dL) was documented and resolved with medical therapy. Wilson disease was excluded by molecular genetic testing of the *ATP7B* gene.

After an uncomplicated spontaneous delivery in 2020, the disease course became progressive, with increasingly frequent episodes of hyperammonemic encephalopathy and worsening gastrointestinal symptoms. The temporal association with pregnancy is noteworthy, as the postpartum deterioration may reflect the haemodynamic changes of pregnancy—including increased splanchnic and portosystemic shunt flow—superimposed on an already marginal decompressive pathway. Abdominal contrast-enhanced computed tomography (CT) in 2024 once again confirmed the absence of the portal vein, and transient elastography showed no fibrosis or steatosis (F0/S0). Despite therapy with L-ornithine-L-aspartate, rifaximin, and regular cleansing enemas, the patient clinically decompensated from January 2025, with recurrent episodes of stupor and coma and recurrent intestinal obstruction. In February 2025, CT showed marked dilatation of both the small and large bowel without any convincing mechanical cause. In May 2025, an emergency laparotomy was undertaken for a further episode of obstruction with free intra-abdominal gas; however, neither a mechanical level of obstruction nor a source of the free gas could be identified, consistent with a dynamic (functional) character of the obstruction. Even during periods of maximal neurological improvement, the baseline ammonia level remained persistently above 110 μmol/L, indicating refractory hyperammonemia driven by chronic portosystemic shunting.

Given the progressive shunt-related encephalopathy, recurrent episodes of stupor, and the failure of medical therapy, the patient was listed for urgent liver transplantation.

**Figure 2:**
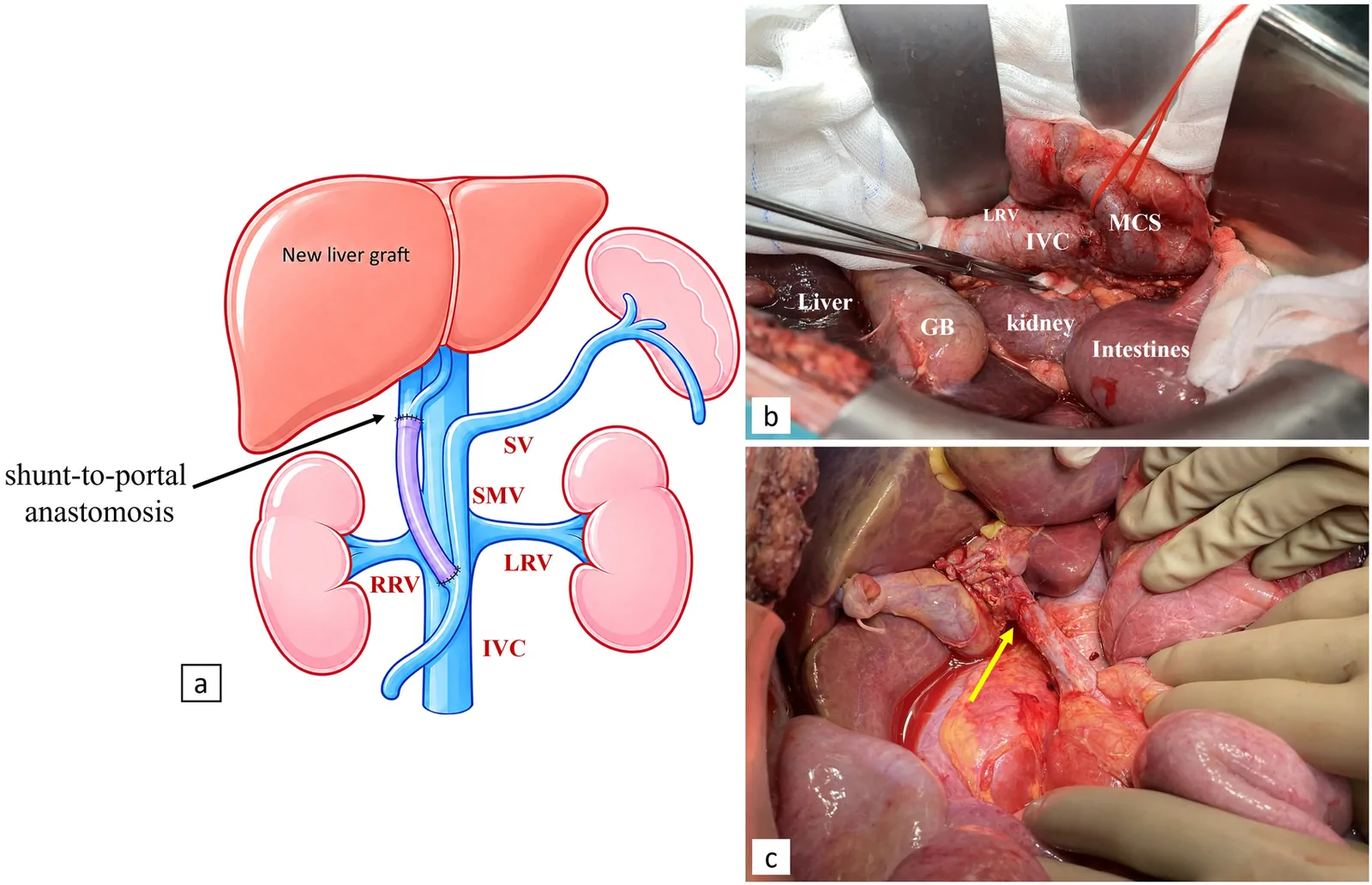
Schematic and intraoperative views of portal inflow reconstruction during orthotopic liver transplantation using a long-standing mesocaval shunt. **(a)** Schematic representation of the portal inflow reconstruction with creation of the shunt-to-portal anastomosis. **(b)** Intraoperative view of the dissected mesocaval shunt and adjacent anatomical structures. **(c)** Intraoperative view after completion of the shunt-to-portal anastomosis (yellow arrow). MCS, mesocaval shunt; IVC, inferior vena cava; SV, splenic vein; SMV, superior mesenteric vein; RRV, right renal vein; LRV, left renal vein; GB, gallbladder.

In July 2025, orthotopic liver transplantation was performed using the Tzakis (piggyback) technique. The graft was obtained from a 26-year-old brain-dead donor who had died of traumatic brain injury; donor liver quality was assessed as satisfactory, with 5% macrovesicular steatosis and laboratory parameters within the reference range. Given the marked bowel dilatation, the procedure began with nasointestinal intubation and small-bowel decompression. During hepatectomy, no native portal vein could be identified. The long-standing H-type mesocaval shunt was selected as the source of portal inflow because it was already mature, of adequate calibre, and suitable for direct anastomosis to the donor portal vein without an interposition graft; this was preferred over dissection of the splenomesenteric confluence or use of a venous conduit, which would have added anastomoses and operative risk in a reoperative field. After the shunt had been mobilized, its calibre and inflow were assessed and judged adequate for graft perfusion, and an end-to-end shunt-to-portal anastomosis was created with the portal vein of the donor liver; adequate portal inflow was confirmed intraoperatively after reperfusion (Figure 2).

The early postoperative course was characterized by the expected ischemia–reperfusion cytolysis with rapid normalization. Peak aspartate aminotransferase (AST) and alanine aminotransferase (ALT) levels reached 891 and 605.1 U/L, respectively, within the first hours after surgery, 549 and 700.6 U/L on postoperative day 1, and 162 and 510 U/L on day 3, and normalized thereafter. Coagulopathy resolved within the same time frame: the international normalized ratio (INR) decreased from 1.39 to 1.15, prothrombin time from 15.1 s to 12.5 s, and prothrombin activity increased from 65% to 86%. Baseline immunosuppression consisted of tacrolimus monotherapy with target trough levels of 6–8 ng/mL, combined with intravenous methylprednisolone with subsequent taper.

On postoperative day 20, the patient developed a cholestatic syndrome (total bilirubin 65.18 μmol/L, direct bilirubin 44.94 μmol/L, gamma-glutamyl transferase 133 U/L, alkaline phosphatase 527 U/L) together with a moderate rise in transaminases. Magnetic resonance cholangiopancreatography (MRCP) revealed a stricture of the biliobiliary anastomosis. On the same day, endoscopic retrograde cholangiopancreatography (ERCP) was performed with endoscopic sphincterotomy and biliary stenting using a fully covered self-expanding metal stent; stent repositioning was required on postoperative day 22. On day 38, the patient was discharged in satisfactory condition, with stable graft function, resolution of dyspepsia, and a regular bowel pattern. Following stent removal on postoperative day 121, recurrent hyperbilirubinemia and imaging signs of restenosis of the biliobiliary anastomosis prompted repeat biliary stenting with a fully covered self-expanding metal stent. The stent was definitively removed on postoperative day 246, and follow-up cholangiography showed no recurrence of the stricture.

Histological examination of the explanted liver showed no cirrhosis. The findings were typical of a congenital portal venous anomaly: absence of intrahepatic portal branches within the portal tracts; reduction in the number of normal portal triads, with formation of arterio-biliary dyads; arterial hypertrophy with an increased number of arterial profiles; and parenchymal vascular remodeling.

**Figure 3:**
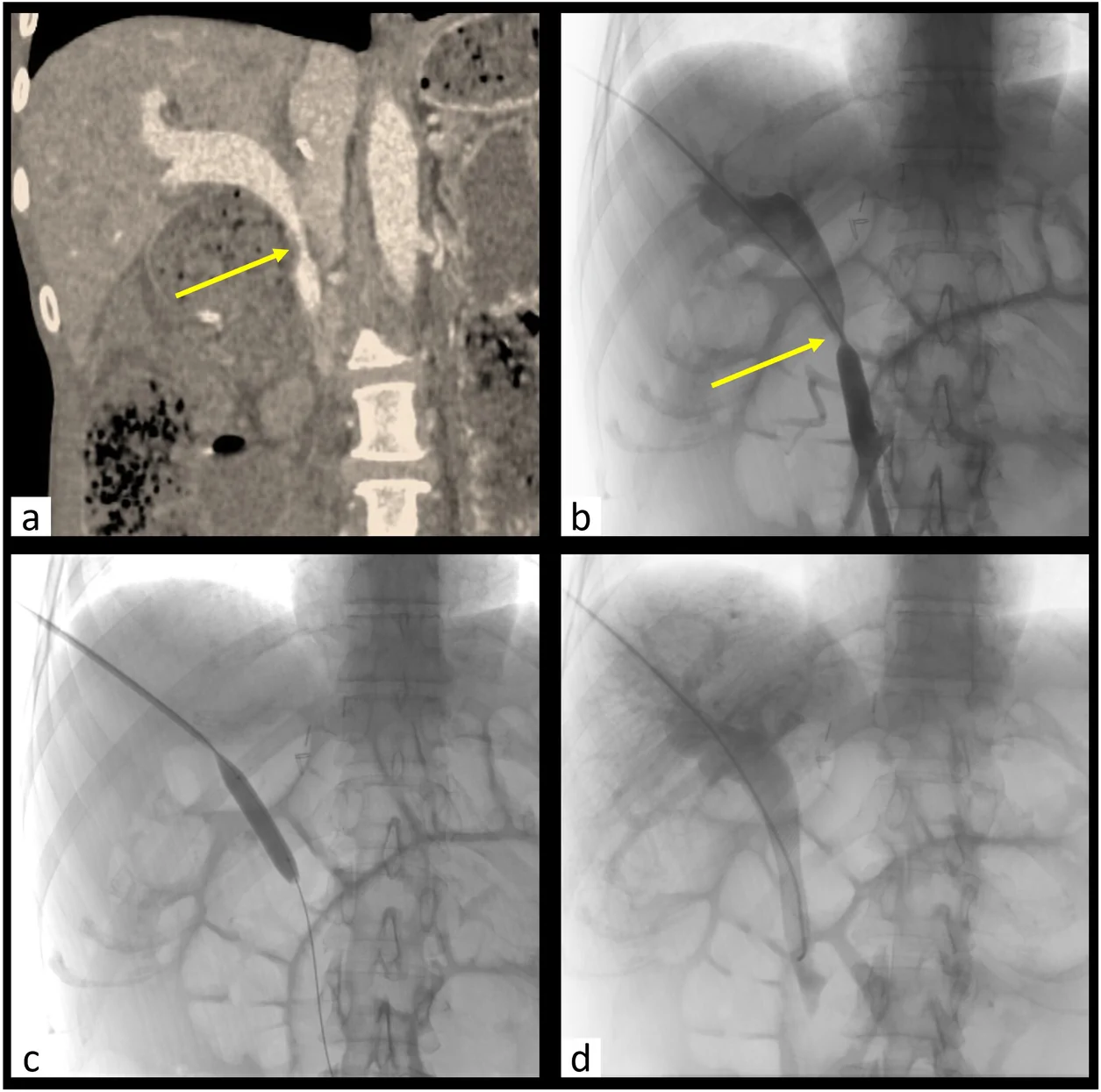
Endovascular management of portal vein stenosis at the shunt-to-portal anastomosis: contrast-enhanced CT **(a)**, pre-intervention portography **(b)**, balloon angioplasty **(c)**, and post-stenting result **(d)** yellow arrows indicate the site of stenosis.

On postoperative day 212, the patient developed new-onset ascites and recurrent vomiting; contrast-enhanced CT demonstrated a stenosis of the portal vein at the shunt-to-portal anastomosis. Endovascular treatment with balloon angioplasty and stenting was performed and led to prompt resolution of the clinical symptoms (Figure 3).

**Figure 4:**
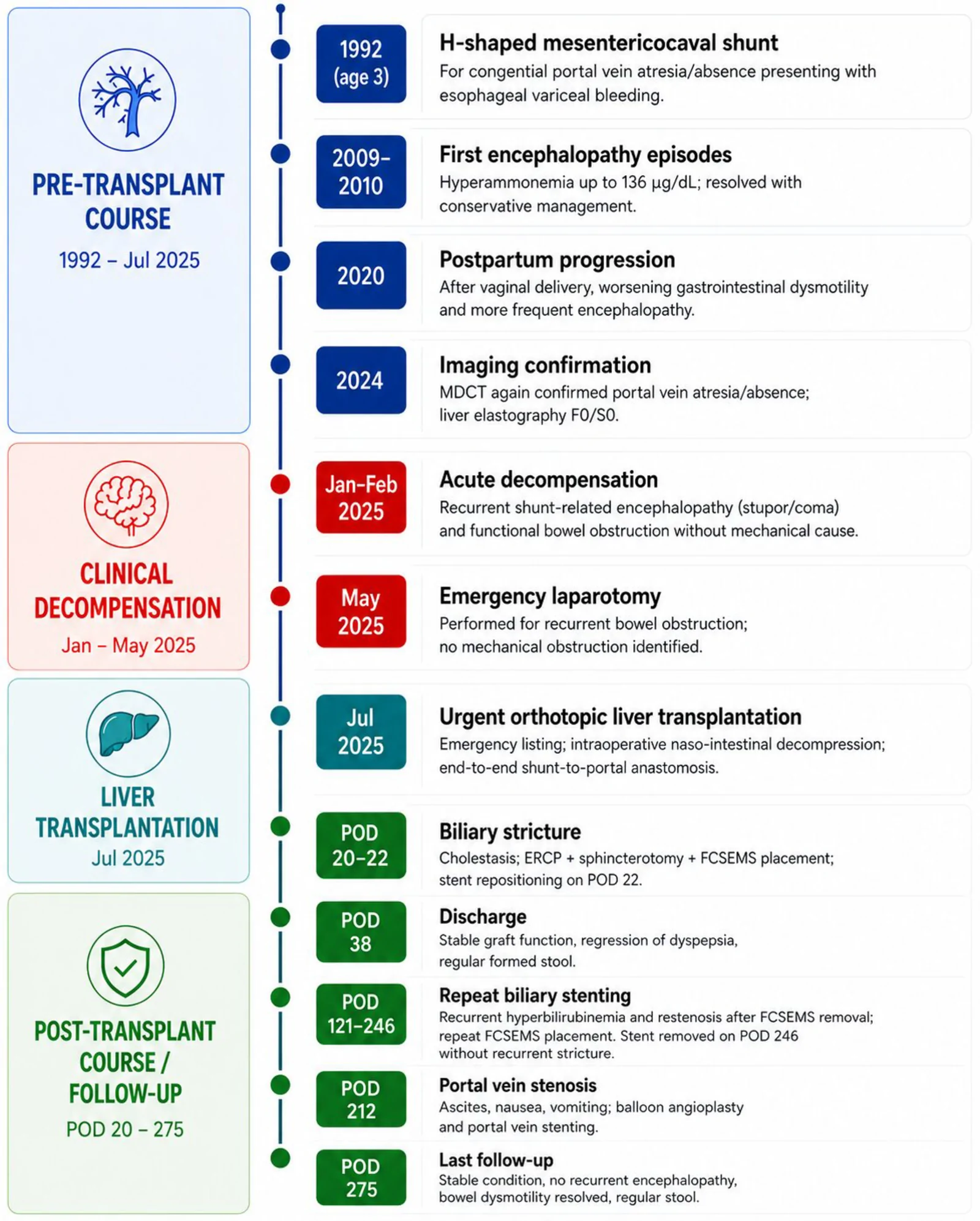
Clinical timeline of the main events in the disease course, surgical treatment, and postoperative follow-up of the patient with congenital absence of the portal vein. The timeline spans childhood presentation with variceal bleeding and H-type mesocaval shunting (1992), the long compensated interval, postpartum clinical progression (from 2020), refractory hyperammonemic encephalopathy with functional bowel obstruction, orthotopic liver transplantation with shunt-to-portal reconstruction (2025), and the subsequent biliary and portal-anastomotic complications with their endoscopic and endovascular management. POD, postoperative day.

At the most recent follow-up (postoperative day 275), the patient remained clinically stable, with no further episodes of encephalopathy, complete resolution of intestinal dysmotility, and a regular bowel pattern. The clinical timeline of the disease course, surgical treatment, and postoperative follow-up is summarized in Figure 4.

## 3 Discussion

This case combines several rarely associated features: atypical hemorrhagic onset of CAPV in early childhood, a latent period of more than three decades after H-type mesocaval shunting, and late decompensation with refractory shunt-related hyperammonemic encephalopathy associated with severe functional bowel obstruction. The surgical interest of the case lies in the use of a long-standing decompressive mesocaval shunt as the source of portal inflow during liver transplantation.

By definition, a functional portosystemic communication is a constitutive feature of congenital portosystemic anomalies rather than an incidental finding (1, 3, 17). In our patient, however, no native congenital shunt could be identified on imaging in adulthood. This does not contradict the diagnosis of CAPV. Two plausible explanations may be considered: the native congenital shunt may have been hemodynamically insufficient from the outset to fully decompress the splenomesenteric venous bed, which would explain variceal bleeding at the age of three years; alternatively, it may have been partially or completely lost over time, including through thrombosis, by the time of clinical presentation (5, 6, 18). Retrospective reconstruction of the original vascular anatomy is not possible because no imaging from that period is available. Therefore, absence of a visible native shunt in adulthood does not disprove CAPV but may reflect either hemodynamic inadequacy or loss of the original decompressive pathway, providing a possible mechanism for the atypical hemorrhagic onset.

The morphology of the explanted liver provides independent support for the congenital nature of the anomaly. The combination of absence of cirrhosis, absence of intrahepatic portal branches, reduction in normal portal triads with formation of arterio-biliary dyads, arterial hypertrophy, and parenchymal vascular remodeling reflects long-term hepatic development in the absence of portal inflow rather than acquired vascular obstruction. This distinction is essential for interpreting the indication for transplantation: the procedure was performed not for end-stage parenchymal liver disease but as definitive correction of the metabolic consequences of a congenital vascular anomaly.

The role of pediatric H-type mesocaval shunting in this case was dual. On the one hand, it provided more than three decades of clinical compensation and effective prevention of recurrent variceal bleeding, in line with historical series (7, 8). On the other hand, mesocaval shunting does not restore hepatopetal flow, and chronic systemic exposure to gut-derived neurotoxic products may culminate in late hyperammonemic encephalopathy. A similar pattern—a long latency followed by metabolic decompensation—has been reported in both pediatric and adult patients with CAPV (11, 12). The present case illustrates that, in patients with a congenitally non-reconstructible portal venous system, a decompressive shunt may delay, but does not necessarily obviate, the need for liver transplantation.

A distinctive clinical feature was severe functional bowel obstruction, which developed in parallel with episodes of hyperammonemia and regressed after transplantation together with the neurological symptoms. Such a complication is not listed among the typical manifestations of congenital portosystemic shunts in current expert reviews (3, 4), which underscores the unusual nature of the present observation. The relationship between intestinal dysmotility and the metabolic state of these patients is biologically plausible: ammonia can modulate enteric neuromuscular transmission through gamma-aminobutyric acid-mediated signaling from enteric glial cells (14), and significant impairment of gastrointestinal motility has been documented in chronic liver disease (15). However, alternative explanations for the regression of bowel symptoms—including resolution of factors related to the long-standing surgical shunt, reduction of chronic inflammatory stimuli, and improvement in nutritional, fluid, and electrolyte status—cannot be excluded in a single case. Notably, bowel function improved after transplantation despite the introduction of tacrolimus, which is itself a recognized cause of gastrointestinal dysmotility and diarrhoea; this argues against a drug-related mechanism and is more consistent with a pre-transplant, metabolically driven process that resolved once portosystemic shunting was eliminated. This interpretation should therefore be regarded as hypothesis-generating.

In the clinical setting described, liver transplantation was the only definitive treatment option. The indication was driven by refractory shunt-related hyperammonemic encephalopathy, failure of medical therapy, and absence of an alternative pathogenetic treatment (3). The main surgical challenge was portal inflow reconstruction. In CAPV, the potential sources of portal inflow are highly variable and dictated by individual vascular anatomy (11, 16). In our patient, the long-standing H-type mesocaval shunt was used for this purpose, making it technically feasible to perform transplantation in the complete absence of a native portal vein and without an interposition venous graft.

Late stenosis of the shunt-to-portal anastomosis became clinically apparent on postoperative day 212 with ascites and recurrent vomiting. A likely contributing factor is the geometric mismatch inherent to this reconstruction: the mesocaval shunt drains inferiorly toward the inferior vena cava, whereas the donor portal vein is oriented superiorly toward the hepatic hilum, and the resulting angulation creates a potential point of traction, rotation, or kinking at the anastomosis. Graft vascular patency was monitored with routine Doppler ultrasonography, and the stenosis was confirmed on contrast-enhanced CT and portography and successfully managed with balloon angioplasty and stenting, followed by antiplatelet therapy. Given this geometry, we suggest systematic Doppler surveillance of the portal anastomosis at fixed postoperative intervals—for example, monthly during the first year—so that hemodynamically significant stenosis can be detected and corrected early; as in the present case, standard endovascular correction may then remain effective.

Overall, the postoperative course supports the chosen strategy. The biliary anastomotic stricture was controlled by staged endoscopic interventions, with stent removal on postoperative day 246 and no subsequent recurrence, and portal vein stenosis was successfully treated endovascularly. At the most recent follow-up, the patient had no signs of encephalopathy and showed resolution of intestinal symptoms, consistent with effective correction of the systemic metabolic consequences of long-standing portosystemic shunting.

## 4 Conclusion

This case broadens the clinical understanding of CAPV by illustrating the possibility of atypical early hemorrhagic onset, several decades of clinical compensation after pediatric decompressive shunting, and eventual metabolic decompensation requiring liver transplantation. The association of refractory shunt-related hyperammonemic encephalopathy with severe functional bowel obstruction, followed by parallel regression after transplantation, suggests a possible shared metabolic mechanism that warrants further study. From a surgical perspective, this case demonstrates that a long-standing H-type mesocaval shunt can serve as a feasible source of portal inflow during liver transplantation when the native portal vein is absent. This reconstructive option expands the technical repertoire for patients with anatomically non-reconstructible portal venous systems and highlights the need for systematic long-term surveillance of the portal anastomosis.

## Patient perspective

Before liver transplantation, my condition gradually worsened despite ongoing treatment. The decision to undergo transplantation was difficult, but I was told that, in my situation, it was the only radical treatment option. I now feel well: the episodes of confusion and severe weakness have not recurred, my bowel function has recovered, my stools have become regular, and I can return to my usual daily activities.

## Data Availability

The original contributions presented in the study are included in the article/Supplementary Material, further inquiries can be directed to the corresponding author.
